# The c.503T>C Polymorphism in the Human *KLRB1* Gene Alters Ligand Binding and Inhibitory Potential of CD161 Molecules

**DOI:** 10.1371/journal.pone.0135682

**Published:** 2015-08-26

**Authors:** Sascha Rother, Joachim Hundrieser, Claudia Pokoyski, Sonja Kollrich, Katja Borns, Rainer Blasczyk, Daniel Poehnert, Jürgen Klempnauer, Reinhard Schwinzer

**Affiliations:** 1 Transplant Laboratory, Department for General-, Visceral- and Transplantation Surgery, Hannover Medical School, Hannover, Germany; 2 Department for Transfusion Medicine, Hannover Medical School, Hannover, Germany; INSERM- CNRS- Univ. Méditerranée, FRANCE

## Abstract

Studying genetic diversity of immunologically relevant molecules can improve our knowledge on their functional spectrum in normal immune responses and may also uncover a possible role of different variants in diseases. We characterized the c.503T>C polymorphism in the human *KLRB1* gene (Killer cell lectin-like receptor, subfamily B, member 1) coding for the cell surface receptor CD161. CD161 is expressed by subsets of CD4^+^ and CD8^+^ T cells and the great majority of CD56^+^ natural killer (NK) cells, acting as inhibitory receptor in the latter population. Genotyping a cohort of 118 healthy individuals revealed 40% TT homozygotes, 46% TC heterozygotes, and 14% carriers of CC. There was no difference in the frequency of CD161 expressing CD4^+^ and CD8^+^ T cells between the different genotypes. However, the frequency of CD161^+^ NK cells was significantly decreased in CC carriers as compared to TT homozygotes. c.503T>C causes an amino acid exchange (p.Ile168Thr) in an extracellular loop of the CD161 receptor, which is regarded to be involved in binding of its ligand Lectin-like transcript 1 (LLT1). Binding studies using soluble LLT1-Fc on 293 transfectants over-expressing CD161 receptors from TT or CC carriers suggested diminished binding to the CC variant. Furthermore, triggering of CD161 either by LLT1 or anti-CD161 antibodies inhibited NK cell activation less effectively in cells from CC individuals than cells from TT carriers. These data suggest that the c.503T>C polymorphism is associated with structural alterations of the CD161 receptor. The regulation of NK cell homeostasis and activation apparently differs between carriers of the CC and TT variant of CD161.

## Introduction

CD161 (NKR-P1A, “natural killer receptor protein 1a”)^3^ is a C-type lectin-like type II trans-membrane receptor which was originally reported to be expressed by natural killer (NK) cells, subsets of αß and γδ T cells, as well as on invariant CD1d specific NKT cells [1–3]. CD161^+^ fractions of CD4^+^ and CD8^+^ T cells have later been identified as producers of the proinflammatory cytokine IL-17 and thus called Th17 [4] and Tc17 [5] cells, respectively, the former originating from CD161^+^ naive CD4^+^ T cord blood precursors [6]. Production of IL-17 has also been described for the CD161 expressing subset among CD4^+^CD25^high^FoxP3^+^ regulatory T cells although these cells have suppressive activity [7, 8] In line with the proinflammatory capacity, Th17 cells are regarded to play a role as pathogenic cells in autoimmune disorders [9, 10] as well as in allograft rejection and vasculopathy [11]. CD161^++^ CD8^+^ T cells have been detected in the cerebral fluid of MS patients [12], and they contain a subpopulation of anti-bacterial T cells termed mucosal-associated invariant T cells (MAIT) [13]. It should be noted, however, that CD161 is not a definite marker for Th/c17 cells. Thus, Fergusson et al. have shown that CD161 expressing T cell subsets are not all committed to the Th17 axis but are in fact much more diverse; yet across all T lymphocyte lineages CD161^+^ subsets share a distinct transcriptional pattern, with an innate-like functional phenotype [14].

Searching for CD161 ligands revealed LLT1 (lectin-like transcript 1) as specific binding partner of the receptor [15, 16]. LLT1 is not expressed on resting cells but is up-regulated by Toll-like receptor (TLR)-mediated activation on plasmacytoid and monocyte-derived dendritic cells (DC) as well as on B cells stimulated through TLR9, surface Ig, or CD40 [17]. Furthermore, T cells can up-regulate LLT1 in response to T cell receptor (TcR)-mediated stimulation [18].

Triggering of CD161 by binding of LLT1 or agonistic antibodies generates inhibitory signals in NK cells. Thus, degranulation and cytokine production induced by stimulation of activatory NK receptors (e.g. NKp46) can significantly be diminished by simultaneous triggering of CD161 [15–17]. Concerning the inhibitory function of CD161 in NK cells, it has been discussed that LLT1 expression by malignant glioma cells could be a mechanism of immune escape preventing effective elimination of tumor cells by innate immune responses [19]. In contrast to the inhibitory potential of CD161 in NK cells, accumulating data have been published showing a co-activating function of CD161 in T cells [2, 12, 14, 15, 18, 20]. It is of note that in immature NK cells from umbilical cord blood CD161 also exerts an activating function [21]. The differential function of CD161 in NK and T cells could indicate a certain plasticity in signaling pathways generating different outcomes depending on cell type, stage of development and/or type of stimulation.

CD161 is encoded by the *KLRB1* gene, which is located on chromosome 12 and is part of the natural killer gene complex (NKC) [22]. In line with other genes of the NKC, CD161 is polymorphic. Several single nucleotide polymorphisms (SNPs) have been described in *KLRB1* and can be found in the Database of Single Nucleotide Polymorphisms (dbSNP, http://www.ncbi.nlm.nih.gov/SNP/). The c.503T>C polymorphism (rs1135816) maps within exon five which encodes part of the extracellular domain of the CD161 receptor. c.503T>C leads to an amino acid-transversion (Ile168Thr) flanking a conserved loop-region [23] of the CD161 receptor which is involved in the binding of LLT1 [24]. Thus, it is conceivable that the c.503T>C polymorphism may influence the capacity of CD161 to bind LLT1, thereby modulating the intensity of signal transduction. Following this assumption, we compared CD161^+^ cells obtained from healthy individuals carrying either the TT or CC genotype of CD161. After triggering of CD161 with LLT1, we observed impaired inhibitory capacity in NK cells from CC carriers. Furthermore, lower frequencies of NK cells in peripheral blood were found. Both observations suggest that c.503T>C is accompanied with functional alterations in CD161.

## Materials and Methods

### Ethics statement concerning blood samples

Blood samples were obtained from healthy voluntary blood donors recruited at the Department of Transfusion Medicine (Hannover Medical School). Cells were isolated from leukotrap filters which are usually discarded after the collection of whole blood. The filters are anonymized/coded and cannot be allocated to an individual blood donor. Thus, written or verbal consent was not obtained. The local ethics committee of Hannover Medical School approved this procedure.

### Human primary cells (PBMC)

Peripheral blood mononuclear cells (PBMC) were isolated from leukotrap filters by Ficoll gradient centrifugation. Cells were kept overnight at 37°C in culture medium (RPMI 1640, supplemented with 10% FCS, 50 U/ml penicillin, 50 μg/ml streptomycin and 4 mM L-glutamin) and then used for genotyping and phenotypic and functional studies. To assess whether overnight storage may influence the subset composition in PBMC, some experiments were performed comparing cell frequencies in freshly isolated cells and cells that had been stored overnight at 37°C. Since we did not find significant differences, all experiments were performed using PBMC after overnight storage.

### KLRB1 mRNA sequencing

To evaluate total genetic variation in exons 1–6 of the human *KLRB1* gene, mRNA was isolated from lymphocytes of c.503T>C genotyped individuals using the RNeasy Mini Kit (Qiagen, Hilden, Germany). Copy DNA (cDNA) was generated through RT-PCR using ImProm (Promega, Mannheim, Germany). KLRB1 cDNA products were amplified and sequenced using primers 5´-GCC TCA CAG AAT TGA AGA GAT GTT TG-3´ (sense), 5´-TCA AGA GTC AGG ATA CAC TTT ATT TC-3´ (anti-sense).

### KLRB1 c.503T>C genotyping

Genomic DNA was isolated from PBMC using QIAamp DNA Blood Mini Kit (Qiagen, Hilden, Germany) according to the manufacturer´s protocol. Concentration and quality of DNA samples were determined by UV absorption (260/280 nm), and DNA was stored at 4°C until usage. Exon 5 of human *KLRB1* was amplified by PCR using primers 161-Ex5-sense (5´-GAT ACA CAC ACA GAA CCT GAT ACG TG-3´) and 161-R2-antisense (5´-CAA ATA AGT TAG TCT ATT TCC TGT C-3´) and 100–250 ng of genomic DNA. Final concentrations were 0.3 μM of each primer, 200 μM dNTPs, 1.5 mM MgCl_2_ in Taq polymerase reaction buffer, and 2U of Taq polymerase (Qiagen) in a total volume of 30 μl. Samples were amplified using 36 cycles in a Perkin Elmer GeneAmp PCR System 9600 (McKinley Scientific, Sparta, NJ, USA). PCR reaction products (3 μl) were controlled by horizontal ethidium bromide-stained 1% agarose gel-electrophoresis. For sequencing, amplification products were purified from PCR reactions by Nucleospin Extract II (Macherey & Nagel, Düren, Germany) according to the manufacturer´s recommendations. Automated sequencing was performed subsequently using sequencing primer (5´-ACA CAG AAC CTG ATA CGT G-3´). Separation and visualization of sequencing products was performed with an ABI PRISM 377 Genetic Analyzer (Life Technologies, Applied Biosystems, Darmstadt, Germany).

### Antibodies and flow cytometry

Antibodies used were obtained from BD Biosciences (San Jose, CA, USA) except where stated. CD161 expression patterns in T cell subsets were characterized by staining PBMC from TT, TC or CC carriers with anti-CD161-bio (B199.2; AbD Serotec, Düsseldorf, Germany) in combination with anti-CD3-PE (HIT3a) and anti-CD4-FITC (RPA-T4), or anti-CD3-FITC (UCHT1) and anti-CD8-PE (RPA-T8). CD161^+^ NK cells were identified by the antibody combination anti-CD161-bio/anti-CD3-FITC/anti-CD56-PE (B159), respectively. Binding of biotinylated antibodies was visualized by a secondary incubation using Streptavidin-Tricolor (Caltag Laboratories, Burlingame, CA, USA). The anti-LLT1-APC mAb FAB3480A was obtained from R&D Systems, Wiesbaden, Germany. Activation induced upregulation of CD107a on NK cells was assessed using anti-CD107a-FITC (H4A3) in combination with anti-CD56-APC (B159) and anti-CD3-PE (HIT3a) or anti-CD3-PerCP (UCHT1; BioLegend, San Diego, CA, USA). Cells were analysed on a FACSCalibur flow cytometer (Becton Dickinson, San Jose, CA, USA) and data were processed by using Summit 5.1 software.

### Cloning of CD161/TT, CD161/CC and LLT1 and generation of 293 transfectants

PBMC from homozygous carriers of CD161/TT or /CC were used for cloning of the two CD161 variants. Total RNA was isolated, reverse transcribed and the resulting cDNA was used as PCR template. RNA from the human B cell line Daudi was used for cloning of LLT1. The following primers with BglII and EcoRI cleavage sites were used for amplification: CD161: sense 5´-GAA GAT CTT CAT GCC ACC TTC CTC TGT CTG-3´, antisense 5´-CGG AAT TCC GTC AAG AGT CAG GAT ACA CTT TAT TTC-3´. LLT1: sense 5´-GAA GAT CTT CCG GCA AAA TGC ATG ACA GTA-3´, antisense 5´-CGG AAT TCC GGA TTA GTT GGG GCT TTG CTG-3´. Samples were amplified by 30 (CD161) or 27 (LLT1) cycles, and the amplified fragments (CD161: 720 bp; LLT1: 630 bp) were digested by BglII and EcoRI restriction enzymes and ligated into the eucaryotic expression vector pIRES2-AcGFP1 (Clontech Laboratories Inc., Mountain View, CA, USA). Vectors recombined with CD161/TT, CD161/TT, or LLT1 and empty vectors (mock) were used for transfection of the human embryonic kidney cell line 293 by Cell Line Nucleofector Kit (Amaxa Biosystems, Lonza Group Ltd., Basel, Switzerland). For the generation of stable cell lines, transfected cells were selected with 2 mg/ml G418. The cells were cloned by limiting dilution and CD161 expression was monitored in individual clones by flow cytometry (anti-CD161-PE; B199.2). Transfectants expressing the same levels of CD161/TT (293-161/TT) or CD161/CC (293-161/CC) were identified and used for further experiments. Additional characterization of CD161 in 293-161/TT and 293-161/CC cells was performed by standard Western-Blotting as previously described [25] using the anti-CD161 mAb B199.2 and anti-α-Actin (AC-40; Sigma, Hamburg, Germany) as loading control. Lysates of PBMC activated for five days with IL-12 (see “CD161 down-regulation on IL-12 activated NK cells”) were used as a further control to estimate the molecular weight of CD161 molecules

### Generation of LLT1-Fc fusion protein

Daudi-derived cDNA was used as template, and a PCR fragment was generated encoding the extracellular domain of LLT1. The following primers with BglII and EcoRI cleavage sites were used: sense 5´-GAA GAT CTT CCG ACA TGT ATA TCT GAT TTG GAA CA-3´, antisense 5´-GGA ATT CGG CAA TAA GAG CTA ACT GCC ATC-3´. The product (402 bp) was double-digested using restriction enzymes BglII and EcoRI and ligated into the eucaryotic expression vector pFuse-hIgG1e2-Fc2 (Invivogen, Toulouse, France). 293 cells were transfected by Cell Line Nucleofector Kit (Amaxa Biosystems) and stable cell lines were selected (culture with 50μg/ml Zeocin). LLT1-Fc protein was purified from supernatants using the Hi Trap protein G sepharose-column and ÄKTAprime HPLC (Amersham Pharmacia Biotech, Piscataway, NJ, USA), and further concentrated using Amicon-Ultra4 filter-columns (Merck, Darmstadt, Germany). Aliquots of protein solutions were stored at -70°C until use and checked for purity and identity by Coomassie Brilliant Blue stained SDS-polyacrylamide gels and immunoblotting, respectively.

### Characterization of the ligand-binding domain of CD161/TT and CD161/CC receptors

The ligand-binding domains were compared between CD161/TT and /CC receptors by studying binding of LLT1-Fc to 293-161/TT and /CC transfectants. The cells were incubated with increasing concentrations of LLT1-Fc and binding of the soluble ligand was monitored by staining with PE-conjugated goat anti-human IgG (Jackson Immunoresearch, West Grove, PA, USA). Furthermore, the “epitope landscape” of the ligand-binding loop was compared between CD161/TT and /CC by studying the affinity of the anti-CD161 mAb 191B8 (IgG2a, Miltenyi Biotec GmbH, Bergisch Gladbach, Germany). Characterization of this mAb and two additional anti-CD161 mAb (DX12, IgG1; B199.2 IgG1, AbD Serotec) revealed that only 191B8 blocks LLT1 binding, suggesting that the 191B8 epitope is part of the ligand-binding domain of CD161. 293-161/TT and /CC transfectants were incubated with an antibody mixture consisting of saturating concentrations (10 μg/ml) of PE-labelled B199.2 and increasing concentrations of unlabelled 191B8. Affinity of 191B8 to this epitope was assessed by studying its capacity to replace/compete with binding of mAb B199.2 which detects an epitope closely related to the 191B8 epitope.

### Assays to evaluate CD161-mediated inhibition of NK cell activation

PBMC from individuals carrying either CD161/TT or /CC were isolated. To induce NK cell activation, 3 x 10^5^ PBMC were stimulated for three hours with 2.5 μg/ml anti-NKp46 (CD337) mAb (9E2, IgG1; BioLegend, San Diego, CA, USA) immobilized on Fcγ R-expressing mouse P815 cells (3 x 10^5^) [26]. The cells were washed and stained with the antibody combination anti-CD107a-FITC (H4A3) / anti-CD56-APC (B159) and anti-CD3-PE (HIT3a) or anti-CD3-PerCP (UCHT1; BioLegend, San Diego, CA, USA). Degranulation/activation of NK cells was determined by assessing the frequency of CD107a^+^ cells in the gated CD56^+^CD3^-^ population. To assess CD161-mediated inhibition of anti-NKp46-triggered NK cell activation, PBMC were stimulated with a mixture of anti-NKp46 (2.5 μg/ml) and anti-CD161 mAb 191.B8 (IgG2a, 5 μg/ml) immobilized on P815 cells. The anti-pig MHC class-II mAb MSA3 (IgG2a; provided by A. Saalmüller, Vienna, Austria) was used as isotype control for the effects of 191B8. % Inhibition was calculated as follows: 100 - [(%CD107a+ NK cells after stimulation with NKp46-CD161 / % CD107a+ NK cells after stimulation with NKp46-isotype) x 100].

In a second approach, NK cell activation was induced by coculturing PBMC (3 x 10^5^) for three hours with 293 cells (3 x 10^4^), which are strong activators of alloreactive NK cell responses [27]. Activation was monitored by assessing CD107a expression on gated CD56^+^CD3^-^ cells. CD161-mediated inhibition was assessed by comparing CD107a expression in gated NK cells after culturing PBMC with 293 transfectants stably over-expressing LLT1 (293-LLT1 cells) and PBMC cultured with 293 mock-transfected cells. To verify that inhibitory effects are solely mediated by the interaction of CD161 on NK cells and LLT1 on transfectants, blocking experiments were performed. To this end, PBMC were pre-incubated on ice with 20 μg/ml anti-CD161 (191B8) or anti-porcine MHC class-II mAb MSA3 and then activated by 293-LLT1 transfectants. % Inhibition was calculated as follows: 100 - [(%CD107a+ NK cells in 293-LLT1 co-cultures / %CD107a+ NK cells in 293-mock co-cultures) x 100].

### CD161 down-regulation on IL-12 activated NK cells

PBMC from individuals carrying either CD161/TT or /CC were isolated. To induce activation and up-regulation of CD161, cells were cultured for five days in standard culture medium supplemented with 2 ng/ml recombinant human IL-12. To assess CD161 down-regulation by CD161-LLT1 interaction, cells were extensively washed, and cultured for three hours, either alone or in the presence of increasing numbers of 293-mock- or 293-LLT1 transfectants (PBMC:293 = 40:1; 20:1; 10:1; 5:1; 2.5:1), respectively. Cells were then spun down and stained with the antibody combination anti-CD3-FITC, anti-CD56-PE, and anti-CD161-APC. % CD161 down-regulation (gated on CD3negCD56pos lymphocytes) was determined as [100 x CD161 MFI (mock)_i_/CD161 MFI (LLT1)_i_]. CD161 MFI of lymphocytes cultured without 293 cells was set as 100%. Index i denominates respective PBMC:293 ratios.

### Statistical analysis

The observed *KLRB1* T503C genotype frequencies in the analysed cohort were comparable to the theoretical distribution according to Hardy-Weinberg equilibrium indicating a likelihood of non-related individuals.

Unpaired t-test or Mann-Whitney *U* test (Graphpad Prism 6 software) was used to compare mean values obtained by two sets of data. Comparison of three data sets (e.g. frequency of lymphocyte subsets in CD161/TT, /TC and /CC individuals) was done using one-way ANOVA and post t-tests. P-values < 0.05 were considered significant (*).

## Results

### Frequency of the c.503T>C polymorphism in healthy individuals

To identify the c.503T>C polymorphism and assess the frequency of different alleles, we sequenced exon 5 of the *KLRB1* gene after PCR-mediated amplification of DNA obtained from healthy blood donors. The T>C transversion in exon 5 could readily be detected at position 503, the result of which is an isoleucin-to-threonine semiconservative amino acid change at position 168 of CD161 (Ile168Thr). Among 118 blood donors we found 47 individuals who were homozygous for the 503T allele (allele frequency 63%) and 17 for 503C (Table 1). In addition to c.503T>C there are 10 entries of SNPs in the dbSNP database (dbSNP, http://www.ncbi.nlm.nih.gov/SNP/) which potentially result in amino acid polymorphisms. Of these, only six had been validated by high-troughput analyses. Two (Glu103Lys, Ser128Gly) with minor allele frequencies of <1%, and four (Asn93Ser, Asn113Ser, Ile139Arg, Arg146His) with frequencies <0.1%. In our cohort we have fully sequenced *KLRB1/CD161* samples obtained from 30 individuals and found no further sequence variations apart from c.503T>C. Thus, we assume that the functional differences between CC and TT individuals presented below are only affected by c.503T>C but not by other variations in CD161.

**Table 1:** Frequency of the CD161 c.503T>C polymorphism in healthy individuals^a^.

Genotype				Allele	
	TT	TC	CC	503T	503C
**Number**	47	54	17	148	88
**%**	39.8	45.8	14.4	63.0	37.0

^a^ The frequency of c.503T>C was determined by analyzing genomic DNA obtained from 118 healthy blood donors. The presence of 503T or 503C was demonstrated by sequencing exon 5 of *KLRB*.

### c.503T>C affects the size and subset composition of the NK cell population

**Fig 1:**
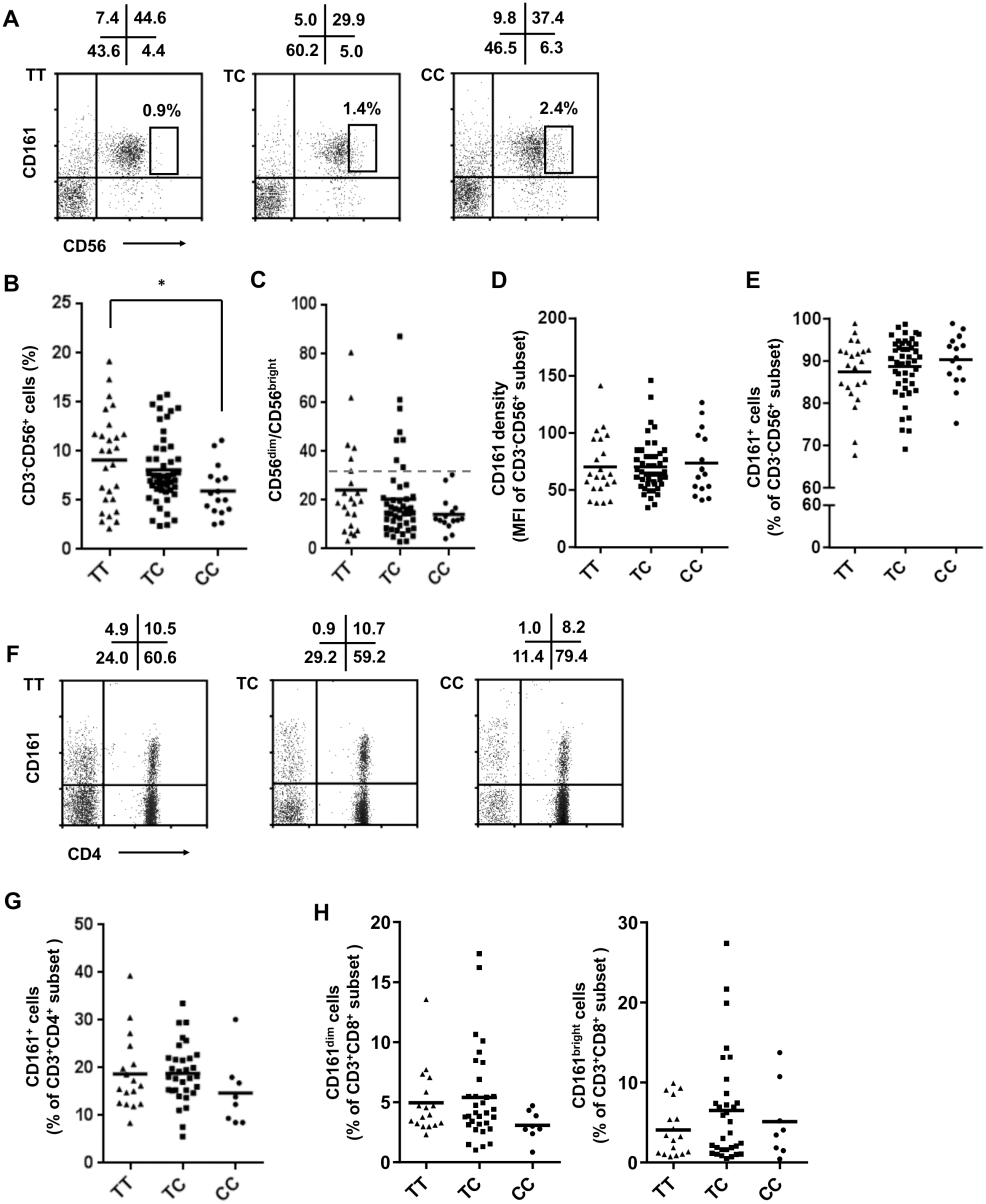
Assessment of lymphocyte subsets in individuals carrying different CD161 genotypes. PBMC were isolated and stained with the antibody combinations CD161/CD3/CD4 or CD161/CD3/CD56. Analyses were performed in gated viable lymphocytes as defined by forward- and side-scatter characteristics. (A) CD161 expression patterns on CD3^-^ NK cells. Representative dot-plots obtained after staining of cells from one TT, TC, and CC individual are shown. Boxes indicate the CD56^bright^CD161^+^CD3^-^ NK cell subset and the frequency. The numbers indicate % positive cells in each quadrant. (B) Decreased frequency of CD3^-^CD56^+^ NK cells in CC carriers. PBMC from 27 TT, 51 TC, and 16 carriers of CC were analyzed. Results are expressed as % NK cells among gated lymphocytes; the mean value calculated for each cohort is indicated by the horizontal line; *p< 0.05 (ANOVA). (C) Ratios of CD56^dim^ to CD56^bright^ NK cells. The frequencies of CD56^dim^CD161^+^CD3^-^ and CD56^bright^CD161^+^CD3^-^ NK cells were determined in gated lymphocytes from 22 TT, 46 TC, and 15 carriers of CC. The horizontal broken line is an arbitrary cut-off value. (D) Level of CD161 expression. Data are expressed as mean fluorescence intensity/MFI on gated CD3^-^CD56^+^ NK cells. (E) Proportion of NK cells co-expressing CD161. Data are expressed as % CD161^+^ cells in gated CD3^-^CD56^+^ NK cells. (F) CD161 expression patterns on CD3^+^ T cells. The numbers indicate % positive cells in each quadrant. (G) Proportion of CD4^+^ T cells co-expressing CD161. (H) Proportion of CD8^+^ T cells co-expressing CD161^dim^ or CD161^bright^.

CD161 expression already occurs in thymocytes [1] and at an early stage of NK cell development [21, 28]. Thus, we assumed that different CD161 genotypes may be associated with variations in CD161 expression levels and/or frequencies of CD161^+^ lymphocytes. To test this possibility, PBMC were isolated from TT (n = 27), TC (n = 51), and CC (n = 16) carriers and stained with the antibody combinations CD161/CD3/CD4 or CD161/CD3/CD56. Because CD161 is expressed almost all NK cells, we first performed a detailed phenotypic analysis of the CD3^-^CD56^+^ subset. These studies revealed that the frequency of CD3^-^CD56^+^ NK cells was significantly reduced in CC carriers compared to TT homozygotes (Fig 1A and 1B). Furthermore, we observed a prominent reduction of CD56^bright^ NK cells in TT individuals compared to CC carriers Quantification of this phenomenon by calculating the CD56^dim^/CD56^bright^ ratios revealed a strong tendency towards reduced ratios in CC individuals (Fig 1C). Although statistical significance was not reached, it was notable that all CC individuals stayed below a ratio of about 30 (Fig 1C, hatched line) whereas several TT and TC carriers had high CD56^dim^/CD56^bright^ ratios. Thus, C503T>C seems to influence not only the size of the NK cell compartment but also the subset composition of the NK cell population. However, the level of CD161 expression on CD3^-^CD56^+^ NK cells and the proportion of NK cells expressing CD161 did not significantly differ between individuals carrying the TT, TC, or CC genotype (Fig 1D and 1E).

Among CD3^+^ T cells, CD161 expression was very similar in TT, TC, or CC individuals and defined two subsets: CD4^+^CD161^+^ and CD4^-^(quasi CD8^+^)CD161^+^ cells (Fig 1F). Detailed analyses of CD161 expression patterns were performed in a cohort of 17 TT, 32 TC, and 8 CC blood donors. These experiments revealed that the proportion of CD4^+^ T cells co-expressing CD161 did not significantly differ between the three genotypes (Fig 1G). Furthermore, among CD8^+^ T cells we did not find significant differences between TT, TC, and CC individuals concerning the proportion of CD161^dim^ or CD161^bright^ cells (Fig 1H).

### Differential binding of soluble LLT1-Fc to CD161/TT and /CC receptor variants

**Fig 2:**
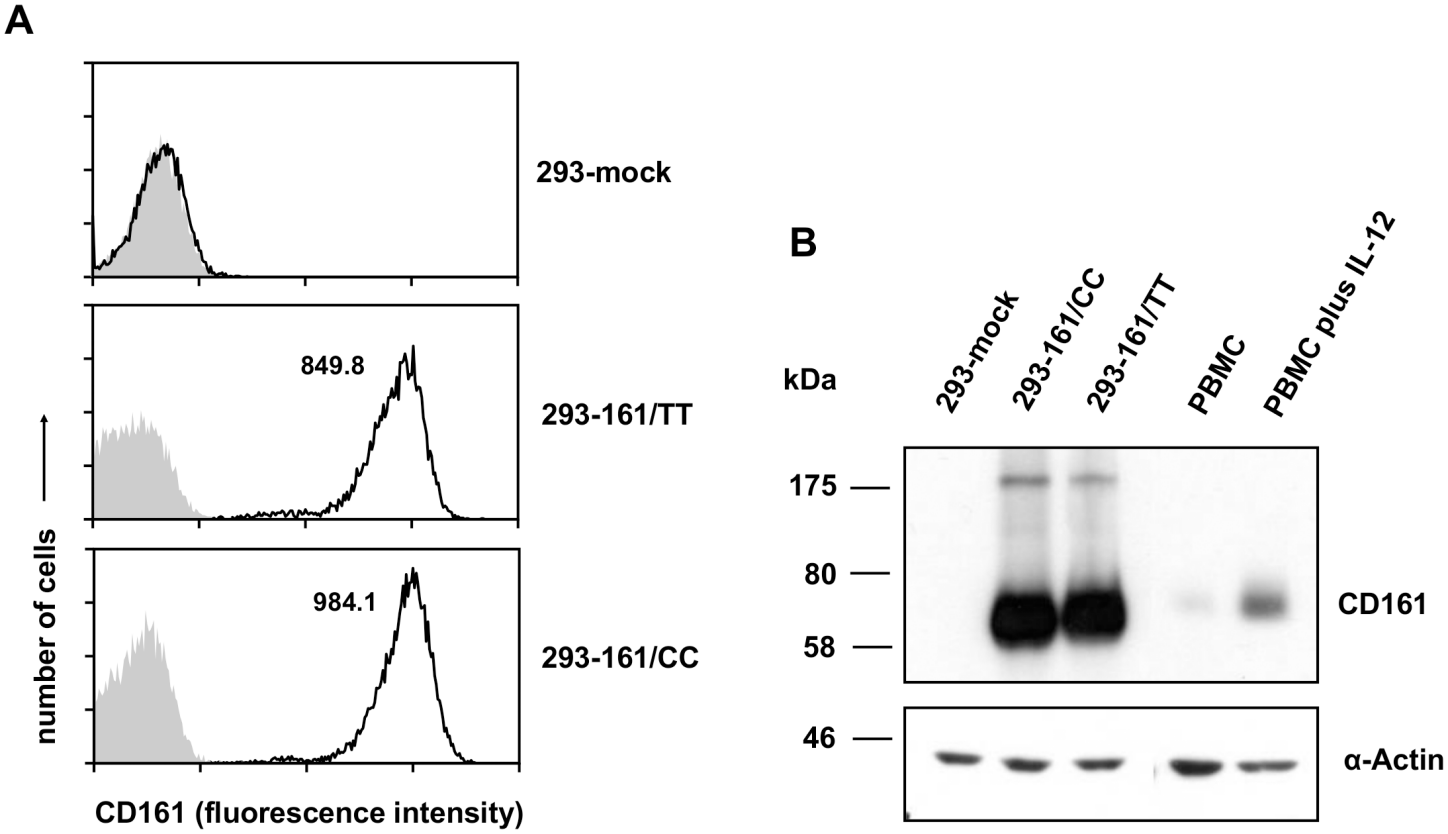
Characterization of 293 transfectants. 293 cells were “mock”-transfected with the empty pIRES2-AcGFP1 vector or vectors containing CD161 from a homozygous TT or CC individual. Analyses were performed after selection of stable transfectants. (A) Flow cytometry analysis of 293-mock, 293-CD161/TT, and 293-CD161/CC cells after staining with the PE-conjugated anti-CD161 mAb B199.2. Grey histograms represent staining with an isotype control mAb. The numbers represent mean fluorescence intensity obtained by anti-CD161 staining. (B) Detection of CD161 by Western-blotting. 293 transfectants were lysed, and protein lysates were separated by 9% SDS-PAGE. After transferring to PVDF membranes, samples were probed with the anti-CD161 mAb (B199.2). Binding of B199.2 was visualized by HRP-conjugated goat anti-mouse-IgG and ECL reagents. Protein loading was controlled by staining with a mAb against α-Actin. Representative results of at least two independent experiments are shown. Lysates of PBMC activated for five days with IL-12 were used as a further control to estimate the molecular weight of CD161 molecules.

**Fig 3:**
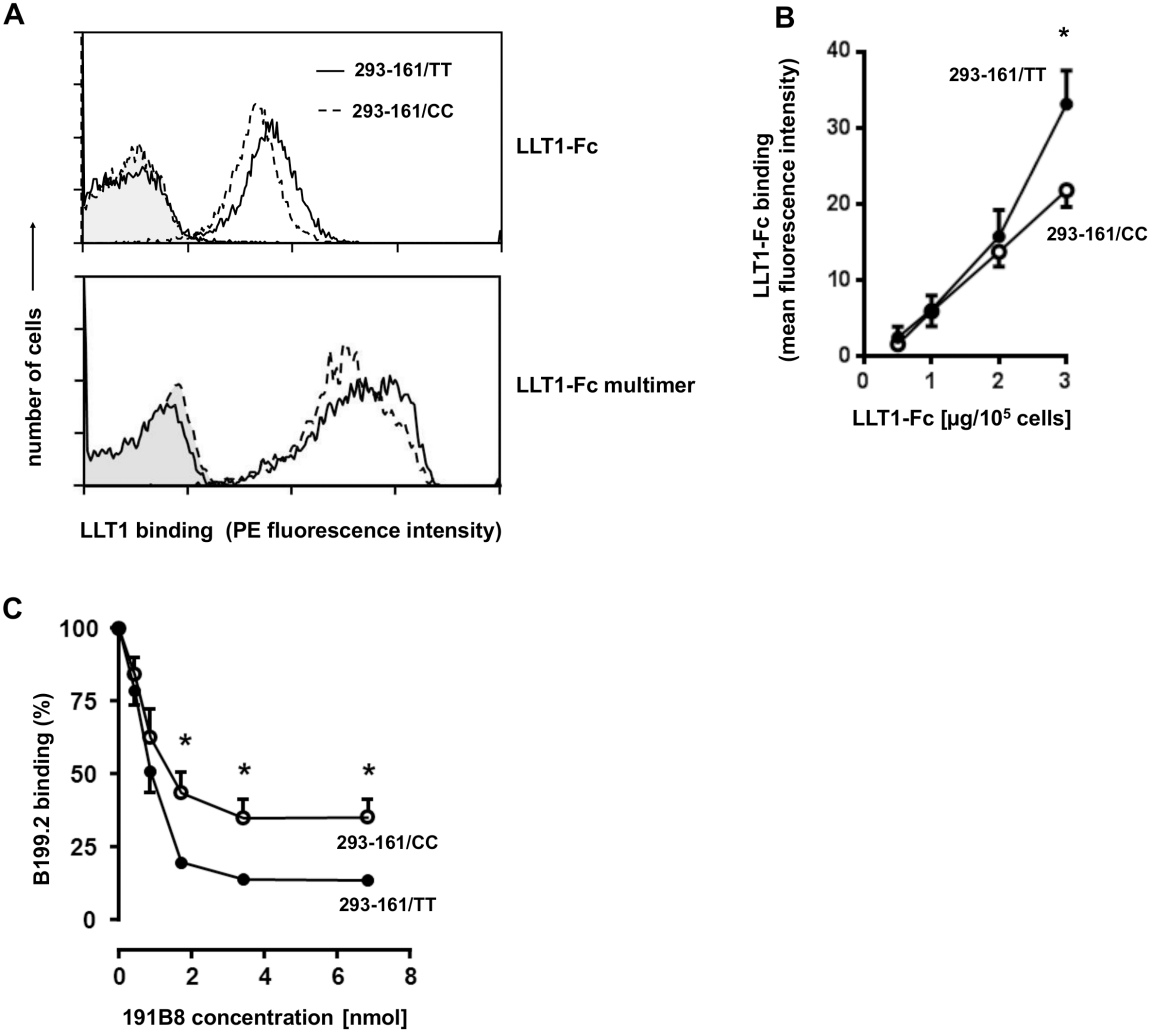
Characterization of the ligand-binding domain of CD161/TT and CD161/CC receptors. (A) Fluorescence histograms obtained after incubation of 293-CD161/TT and -CD161/CC transfectants with control Ig-Fc (closed histograms) and LLT1-Fc or LLT1-Fc proteins multimerized by protein A (open histograms). Binding of the recombinant protein to CD161 was demonstrated by PE-conjugated goat anti-human IgG. (B) Dose-effect relationship of LLT1-Fc binding. 293-CD161/TT and -CD161/CC cells were incubated with increasing amounts of LLT1-Fc. Mean fluorescence intensity (MFI) was determined after a second incubation step with PE-conjugated goat anti-human IgG. Results represent mean values ± SEM calculated for three independent experiments; *p< 0.05 (Unpaired t-test). (C) Epitope-specific antibody competition in CD161/TT and -CC receptor variants. 293-CD161/TT and -CC cells were incubated with a fixed concentration (10 μg/ml) of PE-labelled anti-CD161 mAb B199.2 alone and in combination with increasing amounts of competing anti-CD161 mAb 191B8 (IgG2a) or the anti-pig MHC class-II mAb MSA3 as IgG2a isotype control. Samples were analysed by flow cytometry and PE-MFI was determined. Competition is shown as % binding of mAb B199.2 and was calculated as follows: MFI (B199.2 plus 191B8) / MFI (B199.2 plus MSA3) x 100. MFI obtained with B199.2 in the absence of competing antibody was set as 100%. Results represent mean values ± SD calculated for five independent experiments; *p<0.05 (Unpaired t-test).

The c.503T>C polymorphism causes an amino acid-shift in an extracellular loop structure of the CD161 receptor. Thus, we asked whether TT and CC receptors may have differential binding capacity for the LLT1 ligand. To address this question, a soluble recombinant LLT1-Fc fusion protein was used to label CD161 on T and NK cells from TT and CC homozygous individuals. However, these experiments revealed no clear-cut binding of LLT1-Fc (data not shown). We assumed that low expression levels of CD161 in primary cells could be a reason for the failure to demonstrate LLT1 binding. To overcome this problem, CD161 cDNA was cloned from TT and CC homozygous individuals and 293 transfectants were generated expressing the two receptor variants (293-161/TT and 293-161/CC cells, respectively). Assessment of CD161 expression by flow cytometry and Western blotting revealed strong expression which is almost identical in 293-161/TT and 293-161/CC cells (Fig 2). The molecular mass determined for the CD161 protein coded by the construct resembled that found for CD161 in IL-12 activated PBMC. 293-161/TT and /CC transfectants were incubated with LLT1-Fc and its binding to CD161 was monitored by PE-conjugated goat anti-human IgG, which is directed against the Fc-portion of the recombinant molecule. Histograms obtained after incubation of 293-161/TT cells consistently showed a small but readily detectable shift towards higher fluorescence intensity as compared to staining of 293-161/CC cells (Fig 3A). When the cells were incubated with 3μg of LLT1-Fc, the average value of mean fluorescence intensity was 35 for binding to 293-161/TT and 20 for 293-161/CC cells (Fig 3B). We performed additional experiments using LLT1-Fc proteins multimerized by protein A. Compared to binding of LLT1-Fc there was markedly enhanced binding of LLT1-Fc-ProtA to CD161 transfectants (Fig 3A). However, the high-avidity reagent did not reveal greater differences between TT and CC transfectants than the LLT1-Fc (mean fluorescence TT: 620; mean fluorescence CC: 525).

The data described above indicated that binding of LLT1 is more efficient to TT than to the CC variant of CD161. However, drawing of definite conclusions was somewhat limited since despite using high concentrations of LLT1-Fc, the binding curves did to reach a plateau, supporting earlier observations of weak affinity of the LLT1/CD161 interaction [24]. Therefore, in another set of experiments we compared the “epitope landscape” of the ligand-binding domain in CD161/TT and /CC receptors by studying antibody binding. To this end we first performed an epitope mapping of three different anti-CD161 antibodies (191B8, DX12, B199.2) by blocking experiments. The data revealed partial blocking of B199.2 by 191B8, suggesting closely related/overlapping epitopes. Since 191B8 also blocked binding of LLT1 (not shown), it is likely that the epitopes detected by the two mAb are part of the ligand-binding domain of CD161. 293-CD161/TT and /CC cells were incubated with a mixture of a saturating concentration (10 μg/ml) of PE-labelled B199.2 and increasing amounts of unlabelled 191B8 and binding of B199.2 was monitored by assessing PE-fluorescence intensity. In both transfectants, 191B8 diminished binding of B199.2 dose-dependently and maximal inhibition was achieved between two and four nmol of 191B8 (Fig 3C). Compared to undisturbed binding, approximately 65% of B199.2 reactivity was still possible in 293-161/CC cells whereas 191B8 competition decreased binding of B199.2 in 293-161/TT cells by 85%. This pattern indicates higher affinity of mAb 191B8 to its epitope in TT than CC and is compatible with the assumption that the shape/conformation of the ligand-binding loop differs between CD161/TT and /CC receptors.

### Differential capacity of CD161 TT and CC to inhibit NK cell activation

**Fig 4:**
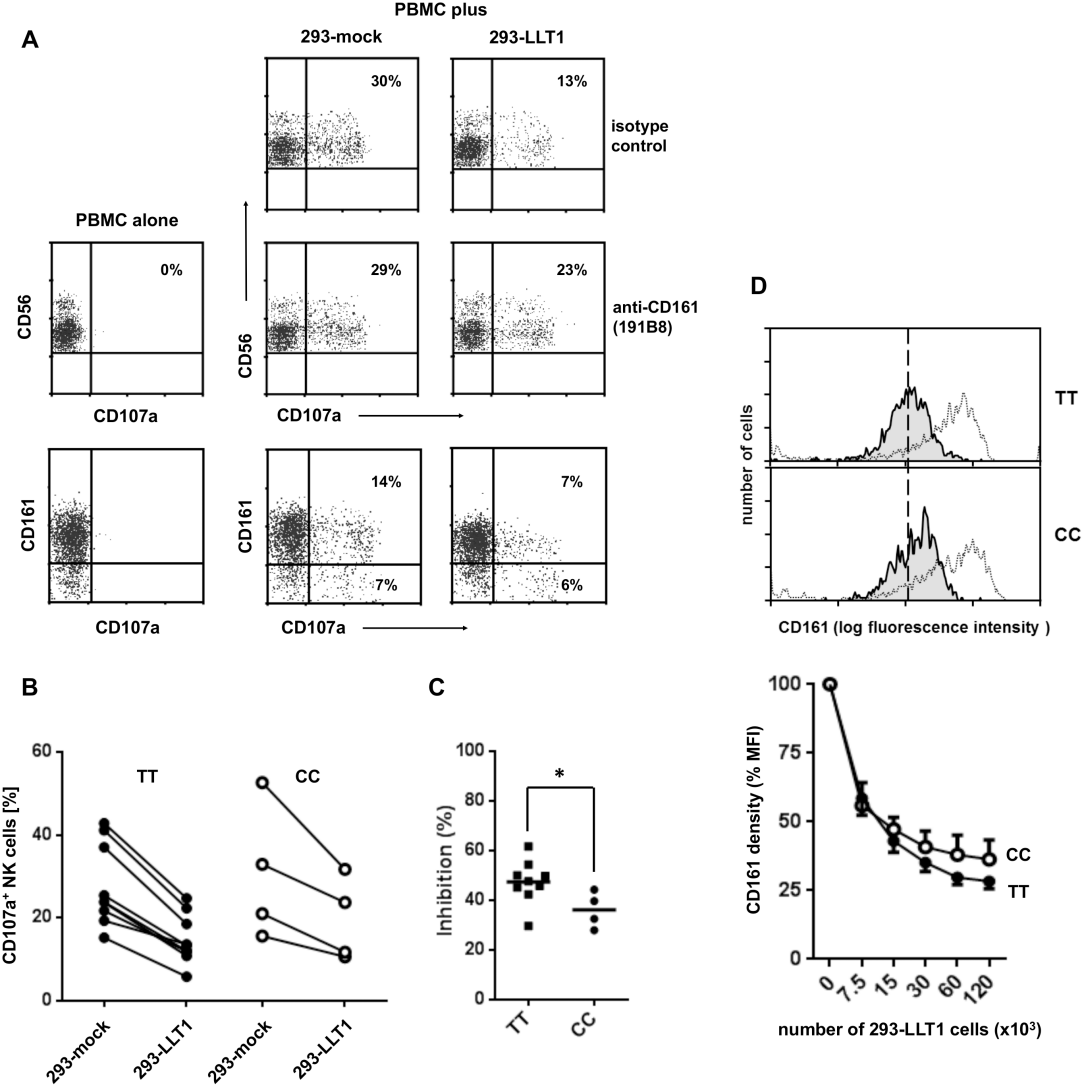
Inhibition of NK cell activation by LLT1-mediated triggering of CD161. PBMC were isolated and co-cultivated for three hours with 293-mock or 293-LLT1 transfectants. Activation of NK cells was assessed by monitoring CD107a expression on gated CD3^-^CD56^+^ and CD3^-^CD56^+^CD161^+^/CD161^-^ cells. (A) Abrogation of inhibition by antibody-mediated blocking of CD161/LLT1 interactions. Co-cultivation of PBMC with 293 transfectants was performed in the presence of the anti-CD161 mAb 191B8 or an isotype-matched control antibody. (B) Frequency of activated NK cells after cultivation with 293-mock or 293-LLT1 cells. PBMC from nine individuals with CD161 TT and four carriers of CC were studied. (C) Diminished capacity of CD161 from CC carriers to inhibit NK cell activation. % Inhibition was calculated as follows: 100 - [(%CD107a^+^ NK cells in 293-LLT1 co-cultures / %CD107a^+^ NK cells in 293-mock co-cultures) x 100];*p<0.05 (Mann-Whitney). (D) LLT1 induced CD161 down-regulation on IL-12 activated NK cells. PBMC were co-cultured for three hours with either mock transfected 293 cells or cells expressing LLT1. CD161 expression was monitored on gated CD3^-^CD56^+^ NK cells. Depicted histograms show CD161 expression in untreated NK cells (open) and after treatment with 293-LLT1 transfectants (filled histograms). The broken vertical line indicates the peak of CD161 observed in LLT1-treated NK cells from a TT individual. The graph summarizes data obtained in experiments using cells from five TT and three CC individuals (mean ± SEM).

Following the hypothesis of differential binding capacity of TT and CC receptors, we asked whether LLT1-mediated triggering of CD161 had different consequences in NK cells from TT and CC carriers. NK cells were activated by co-cultivation with 293-mock controls and 293-LLT1 transfectants and activation was monitored by the emergence of CD107a on the cell surface. Among resting PBMC, CD56^+^ NK cells did not show any signs of activation/degranulation and thus were CD107a-negative (Fig 4A). When PBMC were co-cultivated with 293-mock cells for 3 hours, CD107a was found on a substantial proportion of CD56^+^ cells. In the experiment depicted in Fig 4A, 30% of CD56^+^ NK cells were CD107a-positive. However, when NK cells were co-cultivated with 293-LLT1 transfectants, the proportion of CD107a^+^ NK cells was markedly reduced (13% positive cells). LLT1-mediated inhibition of NK cell activation could be reversed (23% CD107a^+^ cells) by addition of the anti-CD161 mAb 191B8, showing that diminished activation of NK cells is due to CD161/LLT1 interaction. This is also supported by the observation that diminished CD107a expression by 293-LLT1 cells was mainly observed in the CD161^+^ NK subset (14% → 7%) but not in CD161^-^ NK cells (7% → 6%). Using this system, we compared the inhibitory potential of LLT1 on NK cells from CC and TT carriers. As expected, the degree of NK cell activation induced by 293 cells strongly varied between individual blood donors (18 to 52% CD107a^+^ NK cells; Fig 4B). Nevertheless, in all donors we found reduced frequencies of activated NK cells when 293-LLT1 cells were used in the co-cultures. Quantifying the intensity of LLT1-mediated down-regulation of NK cell activation revealed a mean inhibition of 47% in TT and 36% in CC carriers (Fig 4C). Thus, in line with diminished binding of LLT1 to CD161 CC (Fig 3), these data suggest that the intensity of CD161-mediated inhibition of NK cell activation is reduced in CC carriers.

CD161 can be down-regulated by binding of LLT1 [15]. We asked whether the intensity of CD161 down-regulation in response to LLT1 binding differs between cells from TT and CC carriers. To this end PBMC from individuals with CD161 TT and CC were co-cultivated with LLT1 expressing transfectants. Compared to untreated cells, LLT1 binding resulted in down-regulation of CD161 by 71.6±6.1% (mean ± SD) in NK cells from TT individuals (Fig 4D). NK cells from CC carriers down-regulated CD161 only by 63.7±12.2%. Although this difference is statistically not significant, it supports the assumption of diminished binding intensity of LLT1 to CD161 CC.

**Fig 5:**
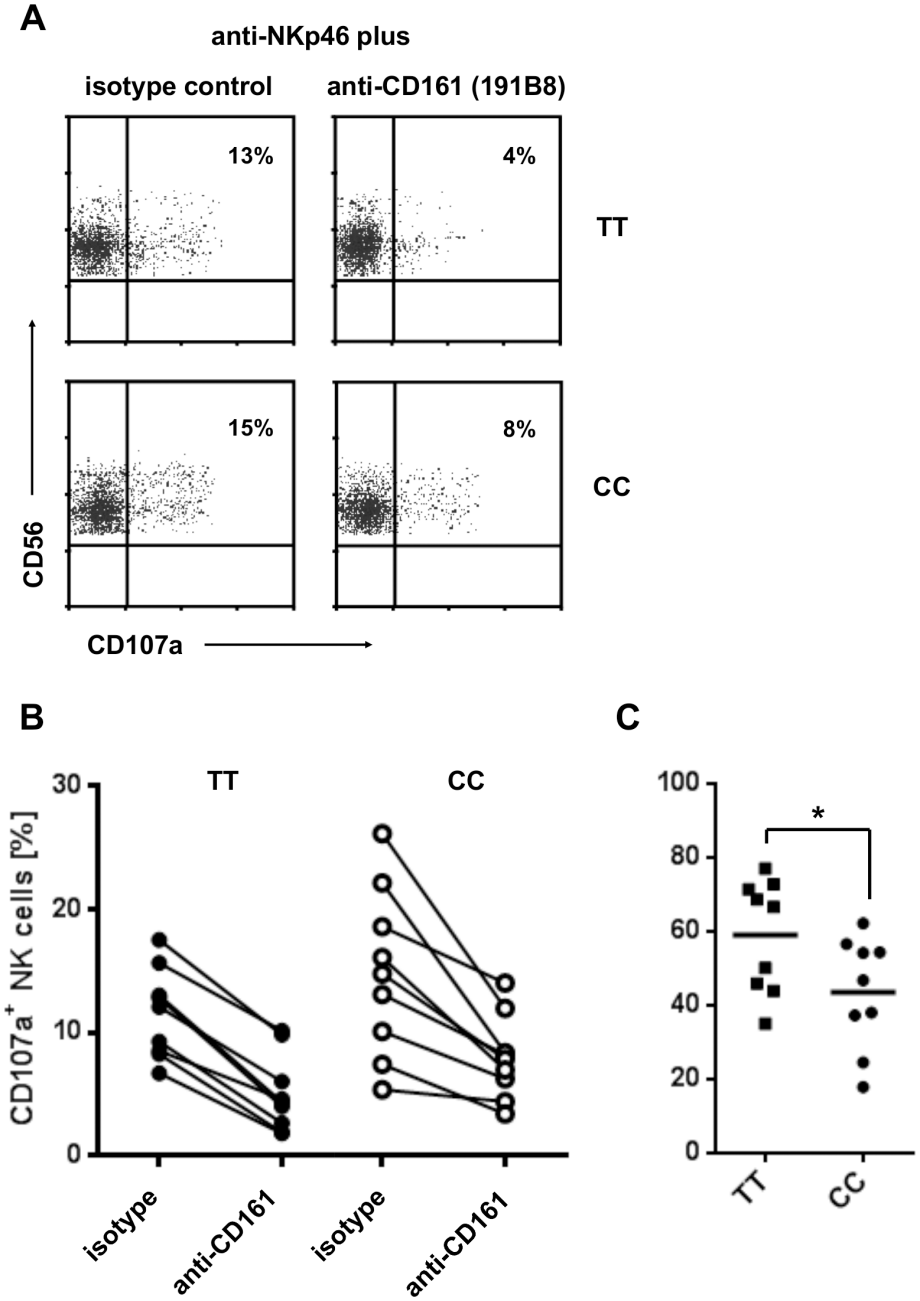
Inhibition of NKp46-induced NK cell activation by antibody-mediated triggering of CD161. PBMC were isolated and stimulated for three hours with the anti-NKp46 mAb 9E2 combined with the anti-CD161 mAb 191B8 or an isotype matched control antibody. The antibodies had been coupled to Fc-receptors on P815 cells. Activation of NK cells was assessed by monitoring CD107a expression on gated CD3^-^CD56^+^ cells. (A) Representative dot-plots showing weaker down-regulation of NK cell activation by anti-CD161 triggering in NK cells from a CC carrier compared to cells from a TT individual. (B) Frequency of activated NK cells after stimulation with anti-NKp46 plus isotype control and anti-NKp46 plus anti-CD161. PBMC from nine individuals with CD161 TT and nine carriers of CC were studied. (C) Diminished capacity of CD161 from CC carriers to inhibit NKp46-induced NK cell activation. % Inhibition was calculated as follows: 100 - [(%CD107a^+^ NK cells after stimulation with NKp46-CD161 / % CD107a^+^ NK cells after stimulation with NKp46-isotype) x 100]; *p<0.05 (Unpaired t-test).

NK cell activation by allogeneic 293 cells is probably mediated through multiple activatory receptor/ligand interactions. Thus, it cannot be distinguished which pathways are inhibited by triggering of CD161. To apply a more defined way of NK cell activation, we used mAb directed to the activatory NKp46 receptor. Cross-linking conditions were achieved by binding of the mAb to Fc-receptors on P815 cells. To assess the effect of CD161 triggering on NKp46-mediated activation, P815 cells were loaded with combinations of anti-NKp46/anti-CD161 mAb or anti-NKp46/isotype-matched controls. As shown in Fig 5A, 13% and 15% of NK cells from TT and CC individuals, respectively, up-regulated CD107a after stimulation with anti-NKp46/isotype control. When activation was performed by the anti-NKp46/anti-CD161 combination, the frequency of CD107a cells decreased to 4 and 8%, respectively. Using this approach, we compared the frequency of CD107a^+^ NK cells in nine TT and nine CC carriers. Again, there was great variability of NKp46-induced NK cell activation between individual donors which, however, could always be diminished by simultaneous triggering of CD161 (Fig 5B). Stimulation of CD161 inhibited NKp46 induced activation in TT carriers by approximately 60% but only by 40% in cells from CC individuals (Fig 5C). The differential effects of antibody-mediated triggering of CD161 in TT and CC NK cells is in agreement with the patterns obtained by LLT1-mediated triggering of CD161 (Fig 4) and suggests weak inhibitory capacity of CD161 CC.

## Discussion

Studying genetic variants of immunologically relevant molecules is one possibility to identify their *in vivo* relevance in the human. CD161 is an inhibitory receptor on NK cells and has been described as marker for a unique subset of T cells. In this study, we characterized the c.503T>C polymorphism in the human *KLRB1* gene. c.503T>C alters the amino acid sequence in an extracellular motif of CD161 which is involved in binding of the LLT1 ligand. We found that the frequency and subset composition of the NK cell population in peripheral blood differs between individuals carrying the CC or TT variant of CD161. This observation supports a role of CD161 in the regulation of NK cell homeostasis and/or migration.

The number of peripheral lymphocytes which can be found in an individual at a given time-point is a function of cell generation/differentiation, survival, and death and also of migration properties of the cells [29]. Based on the observation that the frequency of CD161^+^ NK cells in TT carriers of c.503T>C was significantly increased compared to CC individuals, we assume that CD161 plays a role in this scenario. In contrast to the expression of CD161 on nearly all CD56^+^ NK cells, the majority of CD4^+^ and CD8^+^ T cells is CD161-negative (Fig 1A). A direct involvement of CD161 in the regulation of homeostasis is supported by the fact that differences in peripheral cell frequencies between TT and CC individuals were found in CD161^+^ NK cells but not CD161^-^ T cells (data not shown). CD161 acts as inhibitory receptor in mature NK cells [15–17]. However, in more immature NK cell subsets, triggering of CD161 may generate activatory signals and release of CXCL8 [21]. Anti-CXCL8 neutralizing antibody induced a partial inhibition of NK cell differentiation in that model system suggesting a role of CXCL8 during early NK cell differentiation. Following this scenario, the different numbers of NK cells found in TT and CC carriers may indicate differential signaling of the two types of CD161 receptors resulting in more or less effective NK cell maturation.

Concerning a possible role of CD161 in the regulation of homeostatis, it is worthwhile mentioning that CD161-mediated signalling in NK cells has been described to result in the activation of acidic sphingomyelinase and induction of ceramide [20]. This pathway is regarded to affect apoptosis, proliferation, and differentiation [30]. We also observed an elevated ratio of CD161^+^ CD56^dim^/CD56^bright^ NK cells in several individuals displaying a heterozygous or homozygous TT genotype compared to CC (Fig 1C). The proportion of CD56^bright^ NK cells is differentially distributed between peripheral blood and tissues (e.g. liver, spleen). Thus, altered CD56^dim^/CD56^bright^ ratios depending on the CD161 genotype could indicate a role of CD161 in shuttling of NK cells to/from the periphery [31].

Conclusions that can be drawn from experiments with primary human cells are often somewhat limited because of the heterogeneity of blood donors. Thus, one might argue that the observed differences in NK cell frequencies between TT and CC carriers result from other reasons than c.503T>C. Transgenic mouse models studying this question in detail are not available. However, congenic rat strains which differ only in the CD161-harbouring NKC complex have been described [32]. In our laboratory, NKC-congenic rat strains were developed by backcrossing the NKC from parental strains BS and TO onto the genetic background of LEW. Studying peripheral blood lymphocytes in these strains we found that the frequency of NKRP1A^+^ (rat nomenclature for human CD161) cells co-expressing CD3 (NK-T cells) was significantly different in LEW rats compared to LEW.BS animals (LEW, n = 10: 1.2% ± 0.1; LEW.BS, n = 8: 1.9% ± 0.2; p = 0.0001). This observation is in line with the assumption that genes of C-type lectin like NK receptors clustering in NKC play a role in the regulation of homeostasis.

The hypothesis that differential stimulatory capacity of CD161-TT and -CC receptors in immature NK cells (see above) is the underlying reason for different number of peripheral NK cells in TT and CC carriers could not be tested directly. However, studying the typical inhibitory function of CD161 in mature NK cells from TT and CC carriers indeed suggested differential signalling of the two receptor variants. Inhibition of NK cell activation [33, 34] either by agonistic anti-CD161 antibodies or membrane-bound LLT1 was less effective in CC carriers than in homozygous TT individuals (Figs 4 and 5). Amino acid transversions in ligand-binding domains that also affect receptor function are usually accompanied by altered activation of intracellular signalling cascades [35]. CD161 has been reported to induce ceramide as second messenger, and the activation of Erk-1/2 and PKB/Akt kinases [20]. Due to an intracellular ITIM sequence, several NKR-P1 molecules (e.g. CD161) are supposed to recruit SHP-phosphatases which account for their inhibitory capacity [36]. Based on the diminished inhibitory function we expect to find less effective recruitment of SHP-phosphatases to ITIMs of CD161-CC receptors compared to CD161-TT. Studies are underway to test this possibility.

How can differential signalling of CD161-TT and -CC be explained in molecular terms? Basically there are two possibilities. First, c.503T>C may be associated with different expression levels of CD161 resulting in different number of molecules that are triggered by LLT1 binding in CD161-TT and -CC cells. However, analysis of CD161 expression levels in NK subsets did not reveal significant differences between cells from TT and CC donors, nor did we observe altered receptor expression in CD161^+^ T cells (Fig 1). Second, c.503T>C may be associated with conformational/structural changes in CD161 that affect the affinity of LLT1 binding. To test this possibility, we studied binding of a soluble LLT1-Fc ligand on model cells expressing high levels of CD161-TT or -CC. Binding of LLT1-Fc to CD161-CC was diminished compared with-TT (Fig 3B). Thus, differential ligand binding capacity seems to be more likely the reason for functional differences between CD161-TT and -CC receptors than different expression levels. However it should be noted that SNP-mediated amino acid transversions can alter receptor function by more than one mechanism. In the FcγRIII receptor CD16 a SNP has been described leading to both, higher receptor expression and increased binding of human IgG subclasses [37, 38].

The p.503T>C polymorphism alters the amino acid sequence (Ile168Thr) in a ligand-binding loop-structure of CD161 [24] which is conserved between human and mouse proteins [23]. Evidence that ligand-binding is especially sensitive to sequence mutations in that region have also been obtained in mouse models by showing that one SNP (Ser191Thr) in the binding loop of B6 NKR-P1B (NK1.1) leads to the loss of B6-specific NK1.1 reactivity in BALB/c mice [39]. Our own in silico modelling of the extracellular domains of CD161 TT and CC molecules based on murine homologues suggest that the receptor´s core structure is indeed unaffected by c.503T>C (not shown), yet a mutation in the flexible loop region might affect ligand-induced folding. Especially the latter would even better reflect the % changes observed in ligand-binding and functional capacity of CD161 CC.

The data presented here were obtained by using cells from healthy individuals. Since there were phenotypic and functional differences between NK cells from CC and TT carriers, it is tempting to discuss the question as to whether c.503T>C may act as risk factor for certain diseases. It is of note, that the human *KLRB1* (CD161) gene has been identified as one genetic locus for the risk of developing multiple sclerosis [40, 41]. In the context of autoimmue diseases the diminished inhibitory capacity of CD161 on NK cells from CC carriers could be of relevance. However, since the health status seemed to be similar between TT and CC individuals it is unlikely that c.503T>C SNP on its own is sufficient to cause any severe deviation from normal immune responses. Nevertheless, c.503T>C may act as genetic modifier becoming a risk factors under certain immunological challenges or within a susceptible genetic background. Recently, a subpopulation of FoxP3^+^ regulatory T cells has also been shown to express CD161 and the transcription factor ROR-γt of T_H_17 cells, at the same time sustaining their anti-inflammatory potential [7, 8]. Thus, the impact of the CD161 c.503T>C polymorphism could be complex leading to functional consequences that differ between cell subsets and activation status.

## Supporting Information

S1 Dataset Supporting Information Dataset. (XLSX)
